# Telomere and telomerase in stem cells

**DOI:** 10.1038/sj.bjc.6603671

**Published:** 2007-03-13

**Authors:** E Hiyama, K Hiyama

**Affiliations:** 1 Division of Life Science Research, Natural Science Center for Basic Research and Development, Hiroshima University, 1-2-3, Kasumi, Minami-ku, Hiroshima 734-8551, Japan; 2 Department of Translational Cancer Research, Research Institute for Radiation Biology and Medicine, Hiroshima University, Hiroshima, Japan

**Keywords:** telomere, telomerase, stem cell, cancer stem cell, dyskeratosis congenita

## Abstract

Telomeres, guanine-rich tandem DNA repeats of the chromosomal end, provide chromosomal stability, and cellular replication causes their loss. In somatic cells, the activity of telomerase, a reverse transcriptase that can elongate telomeric repeats, is usually diminished after birth so that the telomere length is gradually shortened with cell divisions, and triggers cellular senescence. In embryonic stem cells, telomerase is activated and maintains telomere length and cellular immortality; however, the level of telomerase activity is low or absent in the majority of stem cells regardless of their proliferative capacity. Thus, even in stem cells, except for embryonal stem cells and cancer stem cells, telomere shortening occurs during replicative ageing, possibly at a slower rate than that in normal somatic cells. Recently, the importance of telomere maintenance in human stem cells has been highlighted by studies on dyskeratosis congenital, which is a genetic disorder in the human telomerase component. The regulation of telomere length and telomerase activity is a complex and dynamic process that is tightly linked to cell cycle regulation in human stem cells. Here we review the role of telomeres and telomerase in the function and capacity of the human stem cells.

Telomere, a complex of guanine-rich repeat sequences and associated proteins, caps and protects every eukaryotic chromosome end against chromosomal fusion, recombination, and terminal DNA degradation (Blackburn, 2001). Telomeric DNA consists of short guanine-rich repeat sequences in all eukaryotes with linear chromosomes, and its length in human somatic cells is remarkably heterogeneous among individuals ranging from 5 to 20 kb, according to age, organ, and the proliferative history of each cell (Wright and Shay, 2005). During a process of DNA synthesis and cell division, telomeres shorten as a result of the incomplete replication of linear chromosomes, the so-called ‘end-replication problem’. This progressive telomere shortening is one of the molecular mechanisms underlying ageing, as critically short telomeres trigger chromosome senescence and loss of cell viability (Collins and Mitchell, 2002; Blasco, 2005; Wright and Shay, 2005). To prevent degradation by exonucleases or processing as damaged DNA, the telomere 3′ single-strand overhang folds back into the D-loop of duplex telomeric DNA to form a protective ‘T-loop’, which is reinforced with TRF2 and other telomeric DNA-binding proteins named Shelterin (de Lange, 2005).

Telomerase is a complex of a reverse transcriptase protein encoded by the *TERT* (telomerase reverse transcriptase) gene and a template RNA TERC (telomerase RNA component). Telomerase can add telomeric repeats onto the chromosome ends, and prevents the replication-dependent loss of telomere and cellular senescence in highly proliferative cells of the germline and in the majority of cancers (Blasco, 2005). Thus, telomerase activity and telomere maintenance are associated with the immortality of cancer cells, germ-line cells, and embryonic stem (ES) cells.

In most human somatic cells except for stem cells and lymphocytes, telomerase activity is diminished after birth so that telomere length shortens with each cell division. A critical length of telomere repeats is required to ensure proper telomere function and avoid the activation of DNA damage pathways that result in replicative senescence or cell death. As stem cells have elongated proliferative capacity, they should have a mechanism that maintains telomere length through many cell divisions. In fact, low levels of telomerase activity have been found in human adult stem cells including haematopoietic and non-haematopoietic stem cells such as neuronal, skin, intestinal crypt, mammary epithelial, pancreas, adrenal cortex, kidney, and mesenchymal stem cells (MSCs) (Table 1). Basically, given the difference of telomere and telomerase activity in human and mouse cells, the telomere and telomerase status in stem cell populations is different between humans and mice (Harrington, 2004). The level of telomerase is low in the majority of human stem cells, whereas it is upregulated in cells that undergo rapid expansion, such as committed haematopoietic progenitor cells, activated lymphocytes, or keratinocytes, even within tissues with a low cell turnover such as the brain (Haik *et al*, 2000; Figure 1).

Mutations in telomerase core components, TERT and TERC, have been found in patients suffering from aplastic anaemia and dyskeratosis congenital (DKC), as described later. Both diseases are characterised by bone marrow failure and/or skin abnormalities, resulting from defects in maintaining the haematopoietic stem cell compartment. Moreover, cancer and ageing, two biological processes in which the role of telomerase has been implicated, are increasingly recognised as stem cell diseases. For example, some cancers may originate from the transformants of normal stem cells or progenitor cells, so-called cancer stem cells (CSCs), whereas ageing is induced through a progressive functional decline in certain stem cells (McEachern *et al*, 2000).

## TELOMERES AND TELOMERASE IN HAEMATOPOIETIC STEM CELLS

In the haematopoietic system, telomerase-expressing cells are stem cells with self-renewal potential and their early descendants (Hiyama *et al*, 1995; Morrison *et al*, 1996). Age-dependent loss of telomeric DNA has been observed in both lymphocytes and neutrophils (Vaziri *et al*, 1993) as well as in other tissues (Hiyama *et al*, 1996; Wright and Shay, 2005). The length of telomeres in adult blood leucocytes was reported to be shorter than that in germline cells from the same donor (Cooke and Smith, 1986). A subset of stem cells isolated from adult bone marrow showed shorter telomeres than a similar subset isolated from fetal liver or cord blood, suggesting that a progressive decline in telomere length with age occurs in haematopoietic stem cells (HSCs) (Vaziri *et al*, 1993). It is possible that transplanted HSCs derived from aged donors may reach their proliferative limit during the lifetime of the recipient. In fact, several cases with graft failure after HSC transplant have been reported to have unusual telomere shortening (Wynn *et al*, 1998).

Low levels of telomerase activity are readily detectable in HSCs and in some of their differentiated progeny including peripheral blood lymphocytes (Hiyama *et al*, 1995; Morrison *et al*, 1996). Nevertheless, the age-related loss of telomeric DNA in these cells suggests that telomerase activity in HSCs is insufficient to completely prevent telomere loss. Telomere length alterations have also been noted in myelodysplastic syndromes (MDS) and chronic myeloid leukaemia (CML), where telomere loss in HSCs may be associated with cellular senescence and ineffective haematopoiesis as well as contributing to genomic instability and subsequent leukaemic progression (Ohyashiki *et al*, 2002). These data support the idea that replicative potential of HSCs could be limited by progressive telomere shortening and that telomere length can be used as an indirect indicator of stem cell proliferative history and potential. Bone marrow insufficiency in a telomerase-deficient disease, DKC (Vulliamy *et al*, 2001), may be further evidence of that.

**Figure 1:**
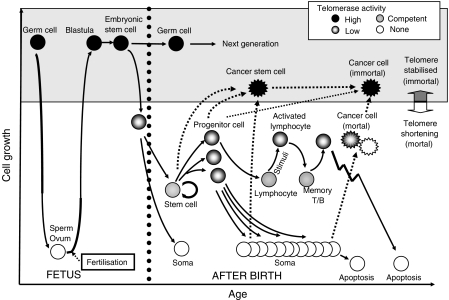
Telomere and telomerase dynamics in human stem cells. Germ cells have high levels of telomerase activity during rapid proliferation. Although telomerase activity is diminished in non-proliferating sperms and ova, it is highly activated after fertilisation and maintained in ES cells and germ cells for the next generation. In the developmental stage, telomerase activity gradually decreases and diminishes in most somatic cells after birth. In adult stem cells, the level of telomerase activity is low or undetectable, and upregulated in committed progenitor cells which have high reproducible activity in each tissue but insufficient to stably maintain their telomere length. Thus, normal stem cells are considered to be mortal and finally senesce by telomere shortening. Cancer stem cells can be derived from normal stem cells, progenitor cells, or possibly somatic cells and might be immortal, having the capacity of indefinite self-renewal and proliferation.

Telomerase activity appears to be upregulated in response to cytokine-induced proliferation and cell cycle activation in primitive HSCs, and progressively downregulated in more matured subsets (Chiu *et al*, 1996) except for mitogenically activated lymphocytes (Hiyama *et al*, 1995) (Figure 1). Stem cells are capable of generating a very large number of committed progenitors and their descendants during a small number of self-renewal divisions. As such, individual stem cell turnover at any given point would be minimal and the upregulation of telomerase activity in stem cells could be minimal, preventing the generation of overly long telomeres (Collins and Mitchell, 2002). Transient activation of telomerase activity induced during periods of rapid cellular proliferation could reduce telomere loss in lineage-committed progenitor cells. In more mature cells, telomerase activity is repressed irrelevant to their proliferation. These findings suggest that one important function of telomerase in HSCs is to reduce the rate of telomere loss during the period of rapid cell division, preventing premature critical shortening of telomeres and loss of telomere function. Such a role of telomerase would be critically important in the case of increased proliferative demand, such as infection or blood loss (Allsopp *et al*, 2003).

## NON-HAEMATOPOIETIC STEM CELLS

Some controversial data remain in telomerase activity and TERT expression in non-HSC (NHSCs). The difference of the levels of telomerase activity in NHSC components of various organs is likely derived from the difference of their cellular turnover among organs. In this review, as representative NHSCs, we focused on MSCs.

Stem cells for mesenchymal tissues, which can be derived from bone marrow, are commonly termed MSCs. Mesenchymal stem cells could differentiate into multiple mesoderm-type cell lineages (Prockop, 1997), such as fibroblasts, adipocytes, osteoblasts, chondrocytes, and endothelial cells, as well as into non-mesoderm-type lineages, such as neuronal-like cells. The phenotype of replicative senescence in cultured MSCs depends on the species. For instance, human MSCs (hMSCs) were reported to cease dividing rather early (around 40–50 population doublings), whereas murine MSCs (mMSCs), that have telomerase activity, could be passaged for more than 100 population doublings (Meirelles Lda and Nardi, 2003). Other investigators also reported for hMSCs that telomere length gradually shortened at a similar rate to non-stem cells (30–120 bp PD^−1^) and telomerase activity was undetectable (Fehrer and Lepperdinger, 2005). However, several growth factors enhance the mitotic potential of hMSC, and we recently found that bone marrow-derived hMSCs maintained long telomeres without the upregulation of telomerase activity for more than 100 population doublings under culture with basic FGF (Yanada *et al*, 2006). Moreover, forced telomerase expression in hMSCs led to an extended life span and enhanced differentiation potential (Simonsen *et al*, 2002). Even using highly sensitive assays, no telomerase activity has been detected in hMSCs so far, and there may be a mechanism of telomere maintenance other than telomerase, such as alternative lengthening of telomeres, in hMSCs. Recent observation of subtelomeric DNA hypomethylation facilitating telomere elongation in mammalian cells suggests that such epigenetic modification of chromatin may occur in hMSCs (Gonzalo *et al*, 2006). On the other hand, mMSCs with their telomerase activity knocked-down completely failed to differentiate into adipocytes or chondrocytes, even in early passages (Liu *et al*, 2004). Also in hMSCs, overexpression of telomerase indeed resulted in the elongation of telomeres, and *TERT-*transfected cells kept proliferating when untransfected control cells ceased growth. Furthermore, the potential of telomerase-overexpressing cells to form bone *in vivo* was greatly enhanced (Simonsen *et al*, 2002). Most likely, telomerase is required for both cell replication and differentiation. The latter implies that hMSCs may express at least the minimum level of telomerase activity to bring about regenerative capacity and differentiation potential.

In other NHSCs, it has been shown that hepatocytes become senescent owing to telomere shortening in the cirrhotic stage of a wide variety of chronic human liver diseases. Intriguingly, mesenchymal cell-like endothelial cells and hepatic satellite cells do not show this replicative senescence (Dan *et al*, 2006). The replicative senescence in hepatocytes is probably in part the result of continued proliferation during 20–30 years of chronic liver diseases. Chronic inflammation, the presence of growth factors, and DNA-damaging agents such as reactive oxygen and nitrogen species may also play a role in this process.

## DISEASES DUE TO LACK OF TELOMERASE IN STEM CELLS: DKC AND APLASTIC ANAEMIA

The critical importance of telomerase activity in human stem cells has been recently highlighted as the aetiology of DKC. In this disease, a defect of the telomerase RNA template gene results in the absence of telomerase activity and premature telomere shortening, developing bone marrow failure, intestinal disorder, or malignancy, typically under 50 years old. Positional cloning identified the gene affected in many patients with X-linked DKC as the human homologue of yeast CBF5, termed *DKC1*, encoding a protein, dyskerin. The association of the telomerase complex with dyskerin suggested that the DKC phenotype may be the result of altered telomerase activity (Vulliamy *et al*, 2001). Then, the discovery of a 3′ deletion in the gene encoding TERC in a single large family with autosomal dominant DKC confirmed the importance of telomerase deficiency as the aetiology for DKC and highlighted the importance of telomerase in maintaining cellular lifespan and replicative potential in human organs. As mice have much longer telomeres that cannot be seriously shortened within their short lifetime, this situation contrasts markedly with *Terc* knockout mice where completely absent telomerase activity is tolerated without abnormality in the first few generations. After the fourth generation, *Terc* knockout mice begin to exhibit abnormalities similar to the phenotype of human DKC (Samper *et al*, 2002). Wild-type mice derived from late generations of Terc +/− mice with short telomeres and positive telomerase activity displayed a similar phonotype of DKC, indicating that short telomeres themselves, rather than lack of telomerase activity, cause stem cell failure (Hao *et al*, 2005). Recently, *Cdkn1a* deletion improved stem cell function and lifespan of mice with telomere dysfunction, indicating that upregulation of p21 (encoded by *Cdkn1a*) in response to shortened telomeres impairs repopulation capacity of stem cells in age-related diseases and senescence (Choudhury *et al*, 2007).

Aplastic anaemia results from the failure of bone marrow to produce sufficient quantities of all haematopoietic lineages. The aetiology of this disease is unclear in most cases, but is generally thought to be the result of HSC damage or loss. Telomere length in peripheral blood granulocytes and monocytes in patients with aplastic anaemia or related disorders was significantly shorter than that in age-matched controls, and correlated with disease duration (Lee *et al*, 2001). An inverse correlation between age-adjusted telomere length and peripheral blood counts was also observed in aplastic anaemia (Brummendorf *et al*, 2001). As in DKC-associated aplastic anaemia, a report found mutations in the *TERC* gene in patients with aplastic anaemia (Vulliamy *et al*, 2002). Data from congenital disorders, like DKC and aplastic anaemia, suggest that disturbed telomere maintenance may play a role in replicative exhaustion of the HSC pool *in vivo*, which might be correlated with age-related diseases and senescence.

## EMBRYONIC STEM CELLS

Embryonic stem cells, more primitive stem cells, are likely to be potentially immortal and capable of indefinite self-renewal together with the ability to differentiate and contribute to the germ line. Embryonic stem cells and undifferentiated embryonal carcinoma (EC) cells display high levels of telomerase activity and hTERT expression, both of which are rapidly downregulated during differentiation (Armstrong *et al*, 2005) and much lower or absent in somatic cells including stem cells in self-renewal tissues (Figure 1). The downregulation of telomerase activity in differentiating EC cells was reported to be tightly correlated with histone deacetylation and DNA methylation of the *TERT* gene (Lopatina *et al*, 2003). Moreover, increased telomerase activity enhanced self-renewal ability, proliferation, and differentiation efficiency in Tert-overexpressing ES cells (Armstrong *et al*, 2005). High telomerase activity or the expression of TERT can therefore be regarded as a marker of undifferentiated ES cells.

In cloned animals originating from adult nuclei with shortened telomeres, telomere length in somatic cells has been found to be comparable with that in age-matched normal animals originating from a fertilised egg with long telomeres. This finding indicates that the enucleated oocyte has the ability to reset the telomere length of the nucleus derived from a donor adult somatic cell by the elongation of telomeres (Meerdo *et al*, 2005). Elucidation of this ‘reset’ mechanism in oocytes will be a technical breakthrough in developing cloned animals.

## CANCER STEM CELLS

The first evidence that a rare population of stem cells maintain the human cancer came from a study on haematopoietic malignancies (Lapidot *et al*, 1994). Haematopoietic malignancies can be divided into premalignant, chronic, and acute stages, the last being the most advanced and malignant. It is known that the premalignant stage, such as MDS, and the chronic stage, such as CML or chronic lymphoid leukaemia, can progress into a more malignant and acute stage of the disease, for example, acute lymphoid or myeloid leukaemia (Greenberg *et al*, 1997). In this progression, telomere shortening in HSCs is considered to be a risk factor that contributes to the development of chromosomal instability and malignant transformation. In agreement with this hypothesis, telomere shortening in patients with DKC correlates with an increased risk of haematological neoplasia (Mason *et al*, 2005). Similar to haematopoietic malignancies, several kinds of solid cancers, especially breast and brain cancers (Singh *et al*, 2003; Al-Hajj and Clarke, 2004), are considered to be generated and/or propagated by CSCs.

To maintain their characteristic replicative ability, CSCs should have telomere-lengthening mechanisms; however, since the identification and purification of CSCs are difficult in most solid tumors, the telomere and telomerase dynamics in CSCs remain unclear. Regardless of the derivation of CSCs, normal stem cells, committed progenitor cells, or differentiated cells (Clarke and Fuller, 2006), CSCs might have acquired immortality by mutational events in telomere-lengthening mechanisms, typically the activation of telomerase (Ohyashiki *et al*, 2002), considering that telomere shortening and cellular senescence are inevitable in these original cells (Figure 1), except for some MSCs. Theoretically, the inhibition of telomerase in CSCs to limit proliferation capacity and induce apoptosis, the stabilisation of telomeres in premalignant lesions to prevent the activation of telomerase and transformation to CSCs, and the identification of specific marker proteins in CSCs in each organ are likely to optimise screening and therapeutic interventions as novel anticancer strategies.

## CONCLUSIONS

Recent research has focused on telomeres and their role in cell cycle regulation, cellular senescence, and genetic instability; however, much remains to be learned, and what is clear is the complexity of telomere and telomerase interactions in cell cycle progression and cell signaling in both normal somatic cells and malignant cells. Both elements have a key role in determining proliferative capacity in all human cells. Telomere shortening occurs in most human somatic cells and triggers DNA damage responses that mediate cell cycle arrest or apoptosis, while HSCs can escape this trigger by employing a telomerase-dependent telomere lengthening mechanism in replication. This process in normal HSCs, however, appears to have only limited potential in extending the replicative capacity, because the telomere shortening occurs slowly but steadily in blood cells with ageing. In contrast, CSCs should have complete telomere-lengthening mechanisms that provide indefinite proliferation capacity. Research on telomere-telomerase dynamics in stem cells could lead to the development of novel aetiology-based radical therapies in ageing-related diseases and cancers.

## Figures and Tables

**Table 1:** Telomere and telomerase in human stem cells

**Cell type**	**Lineage**	**Growth**	**Telomerase**	**Telomere**	**Reference**
Embryonic	All tissue	Unlimited	High	Maintained	
Hematopoietic	Multipotent	Limited	Absent/low	Shortened	Brummendorf and Balabanov (2006)
Mesenchymal	Multipotent/skeletal tissue	Limited/unlimited	Absent/low upregulated by stimuli	Shortened/maintained	(Izadpanah *et al*, 2006; Yanada *et al*, 2006; Zimmermann *et al*, 2003)
Skin	Epidermis	Limited	Low	Shortened	(Sarin *et al*, 2005; Sugihara *et al*, 1999)
Hair follicle	Hair	Limited	Low	Shortened	
Intestinal crypt	Intestinal mucosa	Limited	Low	Shortened	(Hiyama *et al*, 1996)
Neuronal^a^	Several neurons	Limited	Low/absent	Shortened	(Wright *et al*, 2006)
Pancreatic^a^	Ductal epithelial	Limited	Absent	Shortened	(Moriscot *et al*, 2005)
Liver epithelial	Bi-potential	Limited (durable)	Low	Maintained/shortened	(Dan *et al*, 2006)
Cancer stem cell	Multipotent	Unlimited	High	Maintained	(Hertzog, 2006)

a These stem cells may be derived from hMSCs.
